# Supplementary material for: Barriers and facilitators associated with implementing interventions to support oral anticancer agent adherence in academic and community cancer center settings
Source: PLoS One. 2023 Jul 21;18(7):e0286630. doi: 10.1371/journal.pone.0286630 (PMC10361464; doi:10.1371/journal.pone.0286630)
Supplement: Attachment — Submitted filename: PLOSOneReview_Hassan_091422.docx [file pone.0286630.s001.docx]

September 14, 2022,

Dear PLOS ONE Editors:

Thank you for the opportunity to review the manuscript, “Barriers and facilitators associated with implementing interventions to support oral anticancer agent adherence in academic and community cancer center settings.” The manuscript is well written and utilizes an important implementation science framework – the Consolidated Framework for Implementation Research (CFIR) – to identify barriers and facilitators to addressing medication adherence. The application of CFIR allows the authors to group the factors into useful domains that have been shown to be associated with implementation effectiveness. However, my enthusiasm for the manuscript is dampened by the following: there is confusion around comments that refer to the hypothetical intervention and those that refer to the current status quo. I think this can be addressed by dividing the results into current barriers and facilitators to adherence and factors related to implementation of a hypothetical intervention. I also encourage reframing the approach to be more in line with factors thought to be important for implementation effectiveness of an adherence intervention. This may be considered a subtle difference but makes it more inline with the use of the CFIR to design interventions. The CFIR Is an extensive framework and the complete guide quite long. Were all questions and domains included or were some selected and if so that process needs to be included. The other point to make is that a description of the setting is important – which country, average income, access to healthcare services, insurance coverage, etc? I assume this is a US-based study but that needs to be made clear and the context better described.

ABSTRACT:

Methods: remove “known to the research team” doesn’t seem relevant, the acronym “CL” is not defined

Results: see comment above in dividing results to what are perceived current barriers/facilitators and factors associated with implementation effectiveness of an intervention – thinking more of using this to design an intervention. Consider discussing results by domain of CFIR.

Conclusion: “successful implementation” is different from implementation effectiveness. Be careful with use of terminology when using implementation frameworks.

INTRODUCTION

The acronym CL is not defined.

Last paragraph: I think framing this as current barriers/facilitators to adherence and adherence monitoring, plus determining factors that would contribute to implementation effectiveness.

Using a hypothetical intervention is difficult to follow unless the intervention is spelled out in detail (see methods section) and if the person is can fully visualize what that would look like day-to-day. Instead, more useful to frame this as information gathered on what people think would be helpful to see in an intervention to target adherence.

METHODS

The reference is made to prior interventional work on oral cancer therapy management. The introduction section expands on this intervention saying it was effective to some degree but not sustainable. Why were individuals that were part of that intervention not interviewed as opposed to interviewing individuals with a hypothetical intervention they were not involved with? This paper would have been more informative using implementers from the pilot project. If that was not possible that should be explained to provide justification for current sampling. In addition, if information was obtained from the pilot on why it was not sustainable that information should have fed into the interview guide questions and analysis plan.

Study setting: country, income level, characteristics of patients (insurance status, educational level), % current compliance. What are the differences between community and academic center (at organizational, patient, inner/outer setting levels) that justified use of these two settings

Data collection:

Issues regarding using hypothetical intervention as stated above

How were the CFIR constructs that the guide was based on chosen?

RESULTS
Define CL as clinician and AD as administrator

Instead of dividing it into barriers/facilitators may be more helpful to follow if divided into current barriers and factors associated with implementation effectiveness

Table 1: I think dividing things as described above would make things more clear. A brief definition of what each domain and construct refer to would be helpful. The illustrative quotes are helpful but what would be more helpful is a brief description of the emergent theme around adaptability, complexity, etc. Just the word is not helpful when looking at the table.

Table 2 is a good layout but again under barriers there is often just the domain: construct listed not a description or theme that emerged as a barrier. Would be good to have that column remain consistent with the solutions column.

For those not familiar with “external pharmacies” a brief description of this would be helpful.

DISCUSSION:

First paragraph: consider reframing as stated above

Policies/procedures/standardization:

Figure 1: needs a legend, at this time difficult to follow. IS this what happens currently or what the intervention will entail? Or what the hypothetical intervention looks like? I thought the latter but the preceding sentence states this is the aspects of existing OAA, so confusing. Things labeled as moderators seem to be things that may make adherence more or less difficult. Perhaps using alternate terminology to “moderators” such as factors influencing adherence?

Acronyms should be defined
